# Association of Heel Bone Mineral Density With Incident Disability and Mortality in Community‐Dwelling Older Adults

**DOI:** 10.1002/jbm4.10390

**Published:** 2020-08-14

**Authors:** Ryan D Ross, Raj C Shah, Sue E Leurgans, Aron S Buchman, David A Bennett

**Affiliations:** ^1^ Department of Cell & Molecular Medicine Rush University Medical Center Chicago IL USA; ^2^ Department of Orthopedic Surgery Rush University Medical Center Chicago IL USA; ^3^ Rush Alzheimer's Disease Center Rush University Medical Center Chicago IL USA; ^4^ Department of Family Medicine Rush University Medical Center Chicago IL USA; ^5^ Department of Neurological Sciences Rush University Medical Center Chicago IL USA

**Keywords:** ACTIVITIES OF DAILY LIVING, BONE, BONE MINERAL DENSITY, DISABILITY, MORTALITY

## Abstract

Age‐related bone loss is common in older adults. However, the association of low bone mass with incident disability and mortality is not well established. A sample of 738 participants in the Rush Memory and Aging Project (MAP) was evaluated at baseline for bone mineral density (BMD) using quantitative ultrasound at the calcaneus. An annual interview assessed basic activities of daily living (BADL), instrumental activities of daily living (IADL), mobility disability, and history of hip fracture. The associations between baseline BMD and risk of death; incident BADL, IADL, and mobility disability; and hip fracture were investigated using Cox hazard models, adjusting for age, sex, education, race, and body mass index (BMI). The robustness of our findings was evaluated by adjusting for confounding factors and health conditions including joint pain, musculoskeletal medications, smoking status, motor function, global cognition, falls, cardiovascular events, and diabetes. Participants were on average (mean ± SD) 80.9 ± 7.0 years old, 72% female, and 3.8% black, with a baseline BMI of 27.3 ± 5.4 kg/m^2^, and a baseline of BMD of 0.44 ± 0.14 g/cm^2^. In models adjusted for age, sex, education, race, and BMI, lower BMD was associated with a higher rate of death (hazard ratio [HR] 1.20; 95% confidence interval [CI], 1.08–1.33), incident BADL disability (HR 1.20; 95% CI, 1.05–1.37), and hip fracture (HR 2.57; 95% CI, 1.72–3.82), but not of IADL disability (HR 1.00; 95% CI, 0.85–1.17) or mobility disability (HR 1.13; 95% CI, 0.97–1.32). The association between BMD and mortality was not significant in fully adjusted models, but the BMD and BADL associations remained significant in models adjusting for both demographic variables and BMD‐modifying health conditions. BMD is associated with incident disability in older adults. © 2020 The Authors. *JBMR Plus* published by Wiley Periodicals LLC on behalf of American Society for Bone and Mineral Research.

## Introduction

Osteoporosis or low bone mass is a serious public health problem. Recent estimates suggest more than 10.2 million Americans were living with osteoporosis in 2010,^(^ ^1^ ^)^ with an estimated medical cost of $22 billion in 2008.^(^ ^2^ ^)^ Further, over 43.4 million Americans are estimated to have osteopenia,^(^ ^1^ ^)^ a state of low bone mass that is less severe than osteoporosis. Low bone mass can lead to an increased risk of fragility fractures.^(^ ^3^, ^4^ ^)^ Fragility fractures, particularly those of the hip, are associated with reduced functional independence, risk of permanent loss of BADL ability,^(^ ^5^, ^6^, ^7^ ^)^ and high mortality rates.^(^ ^8^ ^)^

Although low bone mass has been established as a risk factor for fractures, which in turn can increase the risk for a number of adverse age‐related health conditions, evidence is emerging that the skeleton influences a number of nonskeletal tissues.^(^ ^9^, ^10^ ^)^ The current study used a sample of community‐dwelling older participants participating in the Rush Memory and Aging Project (MAP) to test the hypothesis that lower bone mineral density (BMD) is associated with risk of death, of incident disability, and of hip fracture. The study utilized quantitative ultrasound (QUS), a portable alternative to the more common dual energy X‐ray absorptiometry (DXA), which has been utilized in cohort studies previously^(^ ^11^, ^12^, ^13^, ^14^ ^)^ and is well correlated to more common clinical DXA measures of BMD.^(^ ^15^, ^16^, ^17^, ^18^, ^19^, ^20^ ^)^

## Subjects and Methods

### Participants

Participants in this study were older, community‐dwelling participants enrolled in the Rush Memory and Aging Project (MAP).^(^ ^21^ ^)^ MAP participants were recruited primarily from continuing care retirement communities in the Chicago, IL, area. Enrollment required no known dementia and consent to brain donation at death. All clinical evaluation, blood draws, and bone density measurements were performed during annual in home visits. The study was approved by an Institutional Review Board of Rush University Medical Center.

Demographic information including date of birth, sex, and years of education were collected via participant interview. Body mass index (BMI) was determined by dividing the measured weight (kg) with the square of the measured height (m).

### BMD

QUS‐based baseline heel BMD measures of the right calcaneus were obtained on a subset of MAP participants between 2002 and 2007 using a Sahara Clinical Bone Sonometer (RM01181; Hologic, Inc., Marlborough, MA, USA). The Sahara system measures the speed of sound (SOS) and the broadband ultrasonic attenuation (BUA), which are used to estimate BMD per the manufacturer's software with an estimated precision of 0.014 g/cm^2^. Performance characteristics and instrument calibration were assessed with routine quality control measurements, which were performed each day before data collection.

### Mortality

Age at death was calculated from date of birth and date of death. Death was determined by date of autopsy, which was conducted on more than 80% of decedents, from quarterly attempts to contact the participants, or via searches of death registries.

### Disability

BADL was assessed annually for up to 15 years after the baseline BMD measurement using a modified version of the Katz scale,^(^ ^22^ ^)^ which includes six basic physical abilities: walking across a small room, bathing, dressing, eating, getting from bed to chair, and toileting. Participants were asked their ability to perform each task with no help, with help, or unable to do so. Participants who reported needing help with or an inability to perform one or more tasks were classified as disabled in BADL.

Instrumental activities of daily living (IADL) was similarly assessed annually and is adapted from the Duke Older Americans Resources Services project.^(^ ^23^ ^)^ Participants were asked to report their need for assistance performing a list of tasks including telephone use, meal preparation, money management, medication management, light and heavy housekeeping, shopping, and local travel. Reponses included the ability to perform these tasks with no help, with help, or unable to do so. Those reporting needing help or unable to perform were classified as having disability in IADLs.

Mobility disability was assessed using the Rosow‐Breslau scale.^(^ ^24^ ^)^ Participants were asked to report whether they required no help, help, or were unable to perform the following activities: heavy work around the house (eg, washing windows or floors), walking up and down stairs, and walking half a mile. For the current study, participants reporting that they required help or were unable to perform any of the three activities were considered to have mobility disability.

### Hip fracture

History of hip fracture is from participant self‐report. Participants were asked whether they had been told by a doctor, nurse, or a therapist that “you had a broken or fractured hip.” Participants were asked annually. The participants with a prior history of hip fracture were identified at baseline and any change in participant response during follow‐up was attributed to hip fractures occurring with the preceding year.

### Motor function

Motor function was assessed using multicomponent performance evaluations, which included composite measures of dexterity, gait, and hand strength.^(^ ^25^, ^26^ ^)^ The motor dexterity score was determined from the composite score of four trials, two trials per hand, using the Purdue pegboard and an electronic tapper (Western Psychological Services, Los Angeles, CA, USA). Motor gait was determined from the time and distance required to walk a distance of 8 feet and to turn 360 degrees twice. Motor hand strength was determined from grip and pinch strength assessments using the Jamar hydraulic hand and pinch dynamometers (Lafayette Instruments, Lafayette, IN, USA). Composite scores for each performance component were obtained by dividing by the sex‐specific median value at baseline, and a global composite was obtained by averaging across the performance evaluations.

### Cognitive function

Global cognitive function was assessed using a battery of 19 cognitive tests designed to measure episodic, working, and semantic memory, as well as perceptual orientation and perceptual speed, described in detail elsewhere^(^ ^21^ ^)^ Briefly, the 19‐test battery included tests for episodic memory (Word List Memory, Recall, and Recognition, immediate and delayed recall of the East Boston Story, Story A from Logical Memory), working memory (Digit Span Forward, Digit Span Backward, Digit Ordering), semantic memory (a 20‐item version of the Boston Naming Test, Verbal Fluency, a 15‐item form of Extended Range Vocabulary, a 20‐item form of the National Adult Reading Test), perceptual orientation (a 15‐item form of Judgment of Line Orientation, and a 17‐item form of Standard Progressive Matrices), and perceptual speed (Symbol Digit Modalities Test, Number Comparison).

### Other variables

Musculoskeletal pain at baseline was assessed by asking participants whether they had pain or aching in any of their joints on most days for at least a month during the prior year.^(^ ^27^, ^28^ ^)^ Individuals who answered affirmatively were subsequently questioned about the location of the pain, which included back or neck, hands, hips, knees, or feet. In these analyses, we used the number of areas reported to be painful as the descriptive variable. Self‐reported physical activity was assessed using a modified version of the 1985 National Health Interview Survey,^(^ ^29^ ^)^ which is reported as a composite measure expressed as the number of hours per week engaged in physical activity. Participant use of musculoskeletal medications, including vitamins, supplements, and over‐the‐counter remedies and medicines, was determined by direct inspection of all medications prescribed by a doctor, including pharmacological treatments for osteoporosis and gout, vitamins, supplements, and over‐the‐counter remedies taken 2 weeks prior to evaluation. Falls were based on participant self‐reporting. Participants were asked “How many times would you say that you have fallen over the past year?” The number of reported falls the preceding year was recorded and the data were compiled as falls present or absent.^(^ ^30^ ^)^ Smoking status was assessed at baseline by asking participants “Do you smoke cigarettes now?” and “Did you ever smoke cigarettes regularly?” Participants were classified as either never smoked, former smoker (does not currently smoke), or current smoker. Circulating calcium level was measured as part of the basic metabolic panel performed by Quest Diagnostics (Secaucus, NJ, USA). Vascular disease burden (0 to 4) was the number of self‐reported history of claudication, stroke, congestive heart failure, and myocardial infarction.^(^ ^31^ ^)^ Diabetes was defined as being present if the participant reported a history of diabetes diagnosis or was taking medications to treat diabetes, as determined by the direct inspection of medication containers.^(^ ^32^ ^)^ Kidney function was assessed using the estimated glomerular filtration rate (eGFR). First serum creatinine was determined by Quest Diagnostics, which was used to estimate the eGFR using the four‐variable modification of diet in renal disease formula.^(^ ^33^ ^)^

### Statistical analysis

To reduce the number of independent variables, we investigated the collinearity between the various QUS‐derived measures by computing Pearson's correlations. All measures showed a high degree of correlation with Pearson's *r* >0.90 for all pairs of measures. Next, we performed a factor analysis including all five QUS measures: BMD, *T*‐score, quantitative ultrasound index (QUI), speed of sound (SOS), and broadband ultrasound attenuation (BUA) to determine whether subsequent modeling could be performed using a single variable. A single variable, BMD, explained nearly 98% of the variability of these measurements and was therefore used as the sole QUS parameter.

An analytical baseline was set at the first BMD measurement. Baseline correlations were assessed using Pearson's correlations on the entire cohort. Men and women were compared with *t* tests, Wilcoxon rank‐sum tests, or chi‐square tests, as appropriate. Discrete‐time Cox proportional hazard models, with terms to control for age, sex, education, race, coded as black versus non‐black, and BMI were used to estimate the cumulative hazard ratio (HR) of incident BADL, IADL, and mobility disability, and hip fracture with BMD as the predictor. Continuous‐time Cox proportional hazard models with similar demographic terms were used to test the association between BMD and mortality. Separate exclusion criteria were applied to the analysis of each outcome, such that participants with that outcome at baseline were excluded. For example, when BADL was the outcome, only participants without a history of BADL at baseline were included in the Cox model; when mortality was the outcome, all participants were included. The models were subsequently augmented with factors likely to influence BMD. A second model was constructed including the interaction between sex and BMD. Next, separate models were run to test the effects of a variety of potential BMD‐modifying health conditions, including vascular disease, kidney function, and diabetic status. Finally, to investigate the influence of hip fractures on BADL disability, we took two approaches to address the following two questions: (i) Does history of hip fracture influence the association between BMD and incident BADL disability? and (ii) Does the development of a hip fracture during follow‐up influence the association between BMD and incident BADL disability? To address the first question, we repeated the fully adjusted Cox model, this time excluding participants with a history of hip fracture at baseline in addition to the exclusion criteria set for each outcome variable. To address the second question, we performed extended Cox models with hip fracture as a time‐varying covariate.

Mixed‐effects regression models were constructed to examine the relationship between baseline BMD and level and annual rate of change in global motor function, each of the composite motor function measures, and global cognition with terms to control for the effects of age, sex, education, and race. Statistical significance was set at alpha = .05. All statistical analyses were programmed in SAS, version 9.4 (SAS Institute, Inc., Cary, NC, USA).

## Results

### Subject characteristics

In total, 738 participants had baseline heel BMD measurements. Baseline demographic characteristics for the sample are presented in Table 1. At baseline, the mean BMD was 0.44 g/cm^2^, which equates to a *T*‐score of −1.25, indicating that the average participant was osteopenic at the time of enrollment. In total, 131 participants were clinically osteoporotic at baseline (*T*‐score ≤2.5), 328 were osteopenic (*T*‐score between −2.4 and −1), and 279 had *T*‐scores greater than −1. A total of 276 participants were classified as former smokers, 24 were current smokers, and 436 had never smoked. Because of the relatively small number of current smokers, current and former smokers were combined for subsequent analysis. Smoking status did not differ by sex.

**Table 1:** Subject Characteristics at Baseline Evaluated (*n* = 738)

Demographic variables	Mean	Female (*n* = 534)	Male (*n* = 204)	Correlation with BMD(Pearson coefficient, *p*)
Age (years), mean ± SD	80.9 ± 7.0	80.8 ± 7.2	81.2 ± 6.5	−0.178, <.01
Female, *n* (%)	534 (72)			
Education (years), mean ± SD	14.5 ± 3.0	14.1 ± 2.7	15.4 ± 3.4^a^	
Race/black, *n* (%)	28 (3.8)	22 (4.1)	6 ± 2.9)	
BMI (kg/m^2^), mean ± SD	27.3 ± 5.4	27.4 ± 5.9	26.8 ± 3.8	0.100, <.01
BMD (g/cm^2^), mean ± SD	0.44 ± 0.14	0.41 ± 0.13	0.52 ± 0.15^a^	
BMD (*T*‐score), mean ± SD	−1.3 ± 1.3	−1.5 ± 1.1	−0.5 ± 1.3^a^	
Use of musculoskeletal medications, *n* (%)	147 (23)	151 (28.3)	9 (4.4)^a^	
Number of painful joint regions (range, 0 to 5), mean ± SD	0.9 ± 1.4	1.1 ± 1.5	0.53 ± 1.0^a^	
Global motor function, mean ± SD	1.0 ± 0.2	1.0 ± 0.2	1.0 ± 0.2	0.240, <.01
Motor dexterity, mean ± SD	1.0 ± 0.2	1.0 ± 0.2	1.0 ± 0.2	0.152, <.01
Motor gait, mean ± SD	1.0 ± 0.3	1.0 ± 0.3	1.1 ± 0.3^a^	0.237, <.01
Motor hand strength, mean ± SD	1.0 ± 0.3	1.0 ± 0.3	1.0 ± 0.3	0.176, <.01
Reported physical activity, mean ± SD	3.1 ± 3.5	2.9 ± 3.5	3.5 ± 3.5	
Global cognition (*Z*‐score), mean ± SD	−0.054 ± 0.6	−0.016 ± 0.59	−0.154 ± 0.70^a^	0.079, .03

Large values for global motor (cognitive) performance correspond to better motor (cognitive) performance.

a Indicates a significant difference between females and males.

At baseline, females had lower BMD scores (*p* < .01) and were more than six times as likely to use musculoskeletal medications (*p* < .01) than males. Baseline BMD was modestly correlated with age. Lower baseline BMD also was correlated with BMI, a lower level of global motor function and its individual components, and a lower level of baseline global cognition.

Overall follow‐up of study participants exceeded 85%. Over an average of 7.3 years of follow‐up, there were 483 deaths. Subsets of the sample were used in subsequent modeling, retaining only participants without baseline disability or hip fracture at the time of analytic baseline. At baseline there 93 participants with baseline BADL, 405 with baseline IADL, 349 with baseline mobility disability, and 41 with a history of hip fractures. In models investigating participants without baseline BADL disability 362 of 593 developed incident BADL, without baseline IADL disability 264 of 311 developed incident IADL, without baseline mobility disability 289 of 351 developed incident mobility disability and without baseline hip fracture history 48 of 626 reported subsequent hip fracture.

### BMD and risk of death

**Figure 1:**
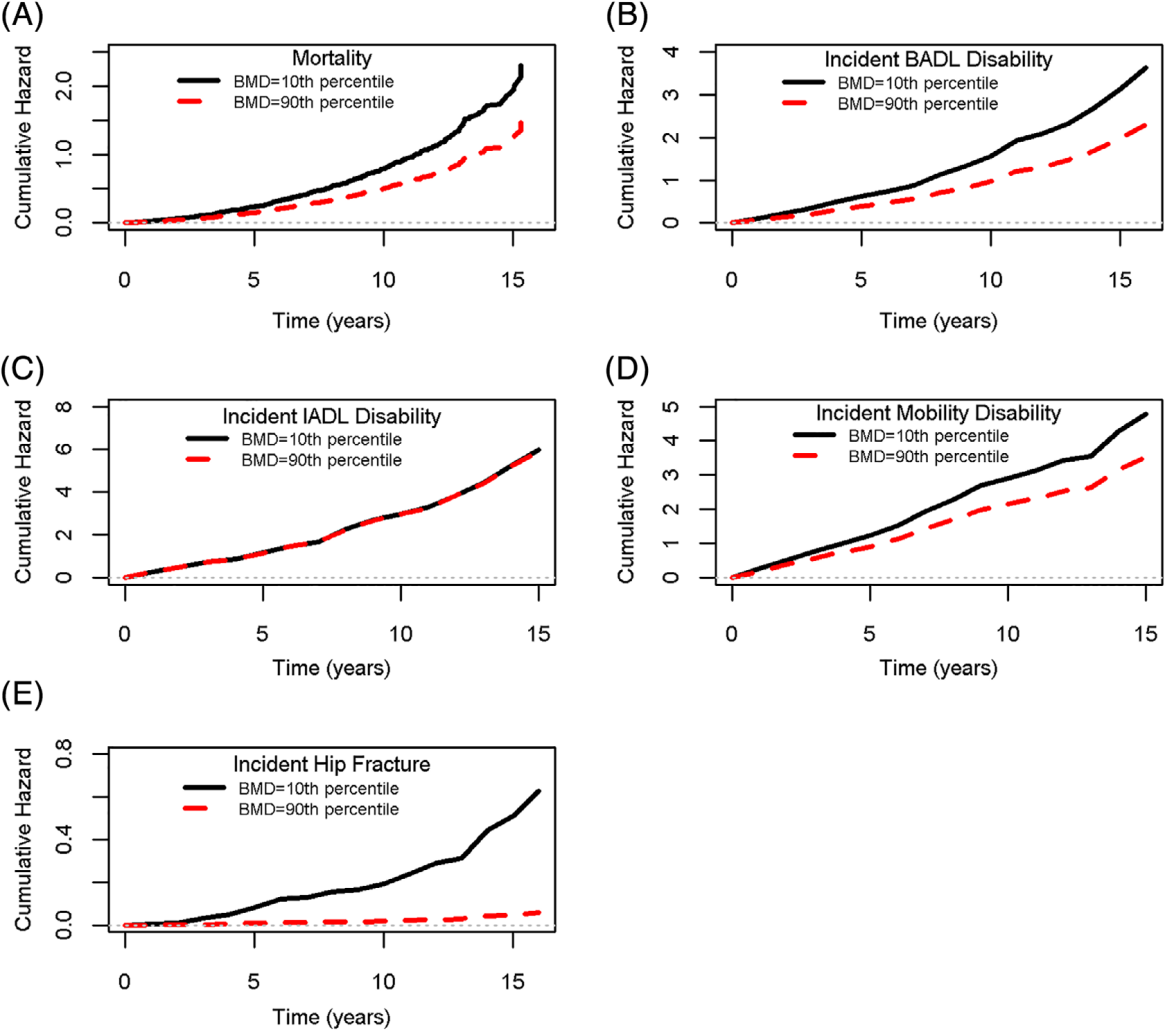
Estimated cumulative hazard functions for (*A*) mortality, (*B*) incident BADL disability, (*C*) incident IADL disability, (*D*) incident mobility disability, and (*E*) incident hip fracture for two hypothetical 80.6‐year‐old female participants with a baseline BMD measure at the 10th percentile (0.27 g/cm^2^) and at the 90th percentile (0.64 g/cm^2^).

We first used Cox proportional hazards models, controlled for age, sex, education, race, and BMI, to assess the relation between baseline BMD and time to death. Lower baseline BMD was associated with a higher hazard rate of death (HR 1.20; 95% confidence interval [CI], 1.08–1.33; Fig. 1*A*). The estimated probability of death within 5 years from baseline was 21.0% for an average participant with baseline BMD at the 10th percentile versus 13.9% for those in the 90th percentile. The elevated risk of death from a baseline BMD 1 SD (0.14 g/cm^2^) lower than the mean is the same as the risk associated with being nearly 2 years older at baseline.

To assess the robustness of the associations, the basic model was augmented to include potential confounding variables and BMD‐modifying health conditions. In fully adjusted models, the association between mortality and baseline BMD was attenuated and no longer statistically significant (Table 2). The BMD by sex interaction term was not significant (*p* = .3) and did not affect the association between BMD and mortality. However, in basic models augmented for various health conditions, such as circulating calcium, vascular disease, diabetes, kidney function, and falls, the association between BMD and mortality was stable (Table 3).

**Table 2:** Association Between BMD and Other Covariates, and Mortality, BADL and IADL Disability, Mobility Disability, and Hip Fracture

Model term	Mortality		BADL disability		IADL disability		Mobility disability		Hip fracture	
	HR (95% CI)	*p*	HR (95% CI)	*p*	HR (95% CI)	*p*	HR (95% CI)	*p*	HR (95% CI)	*p*
BMD	1.10 (0.98–1.23)	.10	1.23 (1.07–1.42)	<.01	0.98 (0.82–1.18)	.86	1.12 (0.95–1.33)	.17	2.67 (1.72–4.13)	<.01
Age	0.93 (0.91–0.95)	<.01	0.97 (0.95–1.00)	.02	0.95 (0.92–0.98)	<.01	0.91 (0.93–0.96)	<.01	0.90 (0.85–0.96)	<.01
Male sex	0.57 (0.45–0.73)	<.01	1.00 (0.72–1.39)	.99	1.68 (1.12–2.51)	.01	1.36 (0.95–1.96)	.09	0.70 (0.30–1.63)	.41
Education	1.00 (0.97–1.04)	.93	0.93 (0.89–0.97)	<.01	1.00 (0.94–1.06)	.89	1.00 (0.95–1.05)	.97	0.97 (0.86–1.10)	.68
Black race^a^	0.92 (0.51–1.65)	.78	2.01 (0.92–4.39)	.08	0.99 (0.41–2.41)	.99	0.93 (0.38–2.28(	.88	—	—
BMI	0.99 (0.97–1.01)	.32	0.96 (0.94–0.99)	<.01	0.99 (0.96–1.03)	.71	0.98 (0.94–1.01)	.19	1.01 (0.93–1.10)	.75
Joint pain	1.04 (0.96–1.11)	.33	0.87 (0.80–0.95)	<.01	0.95 (0.83–1.08)	.41	0.90 (0.80–1.02)	.09	1.20 (0.92–1.55)	.18
Musculoskeletal medications	0.86 (0.68–1.09)	.21	1.01 (0.75–1.35)	.97	1.20 (0.83–1.76)	.34	1.34 (0.94–1.90)	.11	0.75 (0.38–1.50)	.42
Smoking status	0.98 (0.81–1.19)	.83	0.97 (0.75–1.24)	.78	1.09 (0.79–1.51)	.59	1.01 (0.76–1.36)	.93	0.70 (0.38–1.28)	.24
Global motor function	4.99 (2.78–8.95)	<.01	14.12 (6.41–31.15)	<.01	9.00 (3.17–25.58)	<.01	7.32 (2.84–18.90)	<.01	0.47 (0.07–3.26)	.45
Global cognition	1.63 (1.36–1.95)	<.01	2.05 (1.58–2.66)	<.01	2.08 (1.41–3.09)	<.01	1.27 (0.92–1.73)	.14	0.91 (0.48–1.71)	.77
Physical activity	1.01 (0.98–1.04)	.51	1.04 (1.00–1.09)	.04	1.03 (0.98–1.07)	.25	1.02 (0.97–1.06)	.49	1.13 (0.98–1.29)	.10

Results are presented relative to one unit change in the standard deviation of BMD.

a No hip fractures occurred in black study participants; therefore, the model was run in white participants only when hip fractures were the outcome.

**Table 3:** Association Between BMD and Health Conditions and Mortality, BADL and IADL Disability, Mobility Disability, and Hip Fracture

Added model term	Mortality		BADL disability		IADL disability		Mobility disability		Hip fracture	
	HR (95% CI)	*p*	HR (95% CI)	*p*	HR (95% CI)	*p*	HR (95% CI)	*p*	HR (95% CI)	*p*
+ Calcium levels	1.21 (1.06–1.39)	<.01	1.21 (1.06–1.38)	.01	1.06 (0.87–1.29)	.55	1.12 (0.99–1.44)	.07	2.57 (1.48–4.49)	<.01
+ Vascular disease burden	1.19 (1.07–1.33)	<.01	1.20 (1.05–1.37)	.01	1.01 (0.86–1.18)	.94	1.15 (0.98–1.34)	.08	2.55 (1.72–3.80)	<.01
+ Diabetes	1.20 (1.08–1.33)	<.01	1.20 (1.05–1.37)	.01	1.01 (0.86–1.18)	.93	1.15 (0.98–1.34)	.08	2.55 (1.87–3.81)	<.01
+ Kidney function	1.21 (1.05–1.39)	<.01	1.21 (1.05–1.38)	.01	1.00 (0.99–1.02)	.68	1.18 (0.98–1.43)	.09	2.57 (1.48–4.47)	<.01
+ Falls	1.17 (1.05–1.30)	<.01	1.20 (1.05–1.37)	.01	1.00 (0.85–1.18)	.98	1.13 (0.97–1.32)	.10	2.56 (1.78–3.97)	<.01

HR represents the association between baseline BMD and each of the outcome variables after adding the listed model term to the basic model adjusting for age, sex, education, race, and BMI. Results are presented relative to a difference equal to 1 SD of BMD.

### BMD and risk of disability

A parallel set of analyses examined the relation of BMD to BADL disability. Baseline BMD was associated with incident BADL disability (HR 1.20; 95% CI, 1.05–1.37; Fig. 1*B*). The estimated probabilities of developing BADL disability within 5 years were 46.9% and 32.8% for an average participant with a baseline BMD in the 10th and 90th percentile, respectively. The elevated risk of BADL disability from a baseline BMD 1 SD lower than the mean is the same risk as being 2 years older at baseline. The association between baseline BMD and BADL remained significant in fully adjusted models (Table 2) and after adjusting for potential BMD‐modifying health conditions (Table 3). The interaction between BMD and sex was not significant (*p* = .98).

We then examined the relation of BMD to IADL. In basic models, baseline BMD was not associated with incident IADL disability (HR 1.00; 95% CI, 0.85–1.17; Fig. 1*C*). Nor was BMD associated with IADL disability in any of the subsequent models and the interaction between BMD and sex was not significant (*p* = .46).

We next examined the relation of BMD to mobility disability. In basic models, baseline BMD was not associated with incident mobility disability (HR 1.13; 95% CI, 0.97–1.32; Fig. 1*D*). Nor was BMD associated with mobility disability in the fully adjusted models or when evaluated in models adjusting for potential BMD‐modifying health conditions. The BMD by sex interaction was not significant (*p* = .61).

### BMD and risk of hip fracture

Next, we examined the relation of BMD to hip fracture. In basic age, sex, education, race, and BMI adjusted models, low baseline BMD was associated with a higher HR of hip fracture (HR 2.57; 95% CI, 1.72–3.82; Fig. 1*E*). The estimated probably of developing hip fractures was 7.9% and 0.8% for an average participant at the 10th and 90th BMD percentiles, respectively. The elevated risk for hip fracture for 1 SD reduction in BMD is equivalent to being 13 years older at baseline. The association between BMD and hip fracture remained significant in fully adjusted models (Table 2) and after adjusting for calcium levels, circulating calcium, vascular disease, diabetes, kidney function, and falls (Table 3). The BMD by sex interaction was not significant (*p* = .22), nor did it influence the association between BMD and hip fracture.

### Influence of hip fracture on BMD and BADL disability

To assess the influence of history of hip fracture on the association between BMD and incident BADL disability, we re‐ran the fully adjusted Cox proportional model, this time excluding both participants with a history of BADL disability and hip fracture at baseline. The association between BMD and incident BADL remained highly significant in participants without a baseline history of hip fracture (HR 1.22; 95% CI, 1.06–1.41; *p* < .01). We ran similar models with mortality, IADL, and mobility disability as outcome measures and found that the exclusion of participants with a history of hip fracture at baseline had little effect on the associations with BMD (data not shown).

We next sought to determine whether hip fractures mediated the influence of BMD on BADL disability. To do so we first assessed the timing of BADL and hip fractures in the study cohort. In total, 215 participants neither developed BADL disability or hip fractures, 263 developed BADL disability without history of or incident hip fractures, 13 had hip fractures but no BADL disability, and two additional participants reported hip fractures at the last follow‐up visit without history of BADL disability. In the participants that had both BADL disability and hip fractures, 23 had hip fracture first followed by BADL disability, 17 had BADL disability first followed by hip fracture, and 16 participants reported both BADL disability and hip fracture at the same annual visit. In modeling the latter group, we assumed three separate situations: (i) that hip fracture occurred immediately before BADL disability; (ii) hip fracture occurred immediately after the previous visit, roughly 1 year before BADL disability; and (iii) hip fracture occurred after BADL disability. The associations between low BMD and BADL disability remained relatively stable, with HRs of 1.11 (95% CI, 0.98–1.25), 1.12 (95% CI, 0.99–1.26), and 1.14 (95% CI, 1.01–1.28), respectively. However, the significance level was attenuated in models assuming that hip fracture occurred before BADL disability, with the associated *p* values of .09, .07, and .04, respectively.

### Relation of BMD to change in motor function

We next assessed the relationship between baseline BMD and global motor function, as well as, each motor domain, including motor dexterity, motor gait, and hand strength. In age, sex, education, race, and BMI adjusted mixed‐effects models there was an association between baseline BMD and baseline global motor, motor dexterity, and motor gait, but the associations between baseline BMD and rate of change was not significant for any of the motor variable outcomes (Table 4).

**Table 4:** Longitudinal Associations Between BMD and Rate of Global Cognition and Motor Function Decline

Outcome variable	Baseline BMD (95% CI)	*p*	BMD × time (95% CI)	*p*
Global motor function	0.023 (0.001, 0.038)	.04	−0.001 (−0.002, 0.001)	.68
Motor dexterity	0.014 (0.001, 0.027)	.03	0.002 (−0.001, 0.004)	.23
Motor gait	0.040 (0.022, 0.059)	<.01	−0.002 (−0.004, 0.001)	.06
Hand strength	0.008 (−0.012, 0.028)	.45	0.003 (−0.001, 0.006)	.06
Global cognition	0.026 (−0.022, 0.073)	.28	0.006 (−0.004, 0.016)	.25

Mixed‐effects models controlling for age, sex, education, race, and BMI.

### Relation of BMD to change in cognitive function

Because of the strong baseline correlation between BMD and global cognition, we next assessed the relationship between BMD and the rate of change in cognition. In age, sex, education, race, and BMI adjusted mixed‐effects models, we found no association between baseline BMD and the change in global cognition (Table 4).

## Discussion

In the current study of over 700 community‐dwelling elderly persons, low BMD was associated with a greater risk of BADL disability, in addition to its previously established association with a greater risk of hip fractures. The associations were independent of age, sex, education, race, and BMI and remained significant when adjusting for a variety of health conditions, including diabetes and vascular disease. Importantly, the associations were similarly unaffected when adjusting for falls and not fully explained by the development of hip fractures, suggesting that low bone mass may be an independent predictor of BADL disability. The results from the current study extend a growing body of literature suggesting that adverse skeletal health is a critical risk factor for diverse adverse health outcomes.

The current study found a similar cross‐sectional association between BMD and both grip strength and walking speed, included here as part of the motor dexterity outcome, as that noted by Aoyagi and colleagues.^(^ ^34^ ^)^ We have broadened these findings to include longitudinal motor function measurements and found no significant associations between baseline BMD and the rate of change in grip strength and motor dexterity. However, studies in larger cohorts are needed to confirm these results. Although BMD has been linked to physical functioning using a variety of measures^(^ ^14^, ^34^, ^35^ ^)^ including participant‐reported physical activity,^(^ ^36^, ^37^ ^)^ we are not aware of any prior study reporting an association between low BMD and the risk for BADL disability. Although no statistically significant association between low BMD and mobility disability was shown, we speculate that this is due to the high number of participants with mobility disability at baseline, which reduced the power to detect substantial effects. Therefore, further research is needed to determine whether the maintenance of bone mass may protect against the development of more physically demanding mobility disability.

The primary clinical concern for low BMD is the development of fragility or osteoporotic fractures, with fractures of the hip being particularly devastating and associated with considerable economic burden^(^ ^38^ ^)^ and mortality risk.^(^ ^39^ ^)^ Hip fractures are also associated with permanent functional impairment^(^ ^6^ ^)^ and are one potential mechanism linking low BMD to incident BADL disability. In the current study we employed two strategies to test whether hip fractures mediate the association between low BMD and incident BADL disability. The first excluded patients with a history of hip fracture at baseline, because previous fracture is a significant risk factor for future fractures,^(^ ^40^ ^)^ which had no effect on the association. The second approach was to test the influence of the timing of incident hip fracture, which was unfortunately limited by the incomplete resolution on the timing of hip fracture relative to incident BADL disability in the 16 participants who reported BADL disability and hip fracture at the same visit. However, the relatively stable HRs in the time‐varying models suggest the association between BMD and incident BADL is not purely a reflection of hip fractures, pointing to a potential biological link between bone and disability.

There is an increasing understanding that the bone has endocrine function that connects bone and muscle.^(^ ^9^ ^)^ The bone‐derived protein, osteocalcin, for example, has been reported to act on muscle cells to mediate the response to exercise.^(^ ^41^, ^42^ ^)^ In observational studies, osteocalcin levels increase following exercise^(^ ^43^, ^44^, ^45^, ^46^ ^)^ and are positively correlated with muscle strength.^(^ ^47^ ^)^ Therefore, it is possible that bone‐derived signals, such as osteocalcin, are directly contributing to the increased risk of BADL disability and future work is necessary to determine whether osteocalcin serves as a mechanistic link connecting bone and disability.

Low BMD has been reported to be a predictor of mortality risk across a number of studies.^(^ ^11^, ^12^, ^13^, ^48^, ^49^, ^50^, ^51^, ^52^, ^53^, ^54^, ^55^ ^)^ Although the majority of these studies utilized DXA to assess BMD, there have been several studies utilizing QUS of the heel^(^ ^11^, ^13^, ^50^ ^)^ and one that found similar HRs when either DXA or QUS‐derived BMD was used as the predictor.^(^ ^12^ ^)^ A recent meta‐analysis compiled the overall risk and noted a 1.17‐fold increased risk of mortality with 1 SD lower BMD.^(^ ^56^ ^)^ Our study found a similar risk of mortality in models adjusting for age, sex, and BMI; however, when adding additional variables not evaluated in previous studies, such as cognition and quantitative measures of motor function, the association was no longer significant. The attenuation of the association between BMD and mortality in fully adjusted models may suggest mediation by one or more of the covariates included in these models. Future work is needed to determine whether the association between BMD and mortality is mediated by cognition or motor function or whether a larger cohort size is needed to detect a significant association in fully adjusted models.

Strengths of the current study include the large number of confounding variables measured and the use of a cohort of the very elderly (mean age >80 years), the group most at risk for lower BMD.^(^ ^57^ ^)^ We were able to use QUS to fit with in‐home evaluations,^(^ ^58^ ^)^ which correlates with the more commonly performed DXA^(^ ^15^, ^16^, ^17^, ^18^, ^19^, ^20^ ^)^ and is able to predict hip fracture risk.^(^ ^59^, ^60^, ^61^, ^62^, ^63^ ^)^ The limitations of the current study included the incomplete resolution on the timing of hip fracture and the use of participant reported hip fracture, rather than clinical fracture data that would include other skeletal sites, such as the vertebrae. Thus, our study was very limited in its ability to assess whether the association between low BMD and incident BADL disability was mediated by incident fractures among those with low BMD. Another limitation is the lack of longitudinal BMD data, which prevents us from determining whether accelerated bone remodeling with age or a failure to achieve peak bone mass is most predictive of subsequent adverse outcomes. In a study by Cauley and colleagues,^(^ ^64^ ^)^ women with accelerated BMD loss were at a higher risk for mortality and walking difficulty, a finding that would be consistent with elevated bone remodeling. However, their findings suggested that the women who maintained BMD with aging had elevated BMD at baseline; therefore, peak bone mass, which is established decades earlier, may be a possible driver of subsequent age‐related health concerns. Finally, although we included models with a variety of adverse health outcomes, there was no question regarding self‐reported health status, which itself may be a driver of BADL disability.^(^ ^65^ ^)^

The current study investigated the association between baseline BMD and incident disability and mortality, using three different disability measures. We report that low baseline BMD is significantly associated with the development of mortality and incident BADL disability. The associations between BMD and BADL disability remained significant after adjusting for numerous bone‐influencing factors and adverse health conditions, which may suggest that maintaining bone mass is protective of late stage disability. Future work is needed to establish the mechanistic link between the skeleton and the development of age‐related health concerns.

## Disclosures

All authors state that they have no conflicts of interest.

## Author Contributions

**Ryan Ross:** Conceptualization; formal analysis; funding acquisition; investigation; writing‐original draft; writing‐review and editing. **Raj Shah:** Conceptualization; formal analysis; investigation; methodology; writing‐review and editing. **Sue Leurgans:** Conceptualization; data curation; formal analysis; investigation; methodology; writing‐review and editing. **Aron Buchman:** Conceptualization; data curation; funding acquisition; investigation; writing‐review and editing. **David Bennett:** Conceptualization; data curation; funding acquisition; investigation; writing‐review and editing.

## Peer Review

The peer review history for this article is available at https://publons.com/publon/10.1002/jbm4.10390.
